# A comparative experimental study of visual brain event-related potentials to a working memory task: virtual reality head-mounted display versus a desktop computer screen

**DOI:** 10.1007/s00221-021-06158-w

**Published:** 2021-08-04

**Authors:** Murat Aksoy, Chiedu E. Ufodiama, Anthony D. Bateson, Stewart Martin, Aziz U. R. Asghar

**Affiliations:** 1 Centre for Anatomical and Human Sciences, Hull York Medical School, University of Hull, Hull, HU6 7RX UK; 2 Department of Engineering, Faculty Science and Engineering, University of Hull, Cottingham Road, Hull, HU6 7RX UK; 3 School of Education and Social Sciences, University of Hull, Cottingham Road, Hull, HU6 7RX UK

**Keywords:** Virtual reality, Electroencephalography (EEG), Event-related potentials (ERPs), *n*-back, Cognitive workload

## Abstract

Virtual reality head mounted display (VR HMD) systems are increasingly utilised in combination with electroencephalography (EEG) in the experimental study of cognitive tasks. The aim of our investigation was to determine the similarities/differences between VR HMD and the computer screen (CS) in response to an *n*-back working memory task by comparing visual electrophysiological event-related potential (ERP) waveforms (N1/P1/P3 components). The same protocol was undertaken for VR HMD and CS with participants wearing the same EEG headcap. ERP waveforms obtained with the VR HMD environment followed a similar time course to those acquired in CS. The P3 mean and peak amplitudes obtained in VR HMD were not significantly different to those obtained in CS. In contrast, the N1 component was significantly higher in mean and peak amplitudes for the VR HMD environment compared to CS at the frontal electrodes. Significantly higher P1 mean and peak amplitudes were found at the occipital region compared to the temporal for VR HMD. Our results show that successful acquisition of ERP components to a working memory task is achievable by combining VR HMD with EEG. In addition, the higher amplitude N1/P1 components seen in VR HMD indicates the potential utility of this VR modality in the investigation of early ERPs. In conclusion, the combination of VR HMD with EEG/ERP would be a useful approach to advance the study of cognitive function in experimental brain research.

## Introduction

Recent advances in the technical specifications of virtual reality (VR) systems have enabled their utility in the study of cognitive load and function (Cipresso et al. 2018; Luong et al. 2019; Kourtesis et al. 2019; Radianti et al. 2020). Researchers have taken advantage of VR systems in a head-mounted display (VR HMD) configuration in their investigation of cognitive tasks as such systems offer the ability to create/control the visual surround and deliver complex stimuli (Harjunen et al. 2017; Rupp et al. 2019; Dey et al. 2019; Tauscher et al. 2019; Tremmel et al. 2019). Electroencephalography (EEG) is a non-invasive method which conveniently acquires brain neuronal activity in human participants by recording voltage differences at the scalp surface in the millisecond range of temporal resolution (Gevins 1998). Interestingly, studies have shown the feasibility of combining VR HMD with EEG to acquire brain responses to various cognitive tasks: visual oddball (Tauscher et al. 2019), *n*-back working memory (Dey et al. 2019; Luong et al. 2019; Tremmel et al. 2019), 3-choice vigilance and image recognition (Rupp et al. 2019) and bimodal oddball (Harjunen et al. 2017) tasks. In terms of signal-to-noise levels, Harjunen et al. (2017) demonstrated that, despite their a priori concerns regarding signal interference from the VR HMD system upon EEG signals, there was no difference between the results obtained using a VR HMD system and a desktop computer screen (CS).

EEG is also used to acquire event-related potentials (ERPs) which are positive or negative voltage deflections in response to specific time-locked cognitive stimuli or events (Blackwood and Muir 1990; Sur and Sinha 2009). ERP studies have been undertaken in the investigation of working memory using the *n*-back task which requires the continuous maintenance, updating and manipulation of information (Barrouillet et al. 2004; Chen and Huang 2016). The visual *n*-back task involves participants processing stimuli that are presented in a sequence on a screen whilst being asked to determine whether the current stimulus matches a pre-specified *n*-back target (Watter et al. 2001). For example, in the 1-back condition, participants are required to match the current stimulus with the one immediately prior (Watter et al. 2001; Pelegrina et al. 2015; Scharinger et al. 2015).

The most reported and prominent visual ERP component in response to the *n*-back task is a waveform at ~ 300–800 ms post-stimulus termed, the P300 or P3 component (Brouwer et al. 2012; Dong et al. 2015). The P3 component has been implicated to be involved in attention, working memory, task performance, predictability, and relevance judgment (Sutton et al. 1965; Kahneman 1973; Ruchkin and Sutton 1978; Picton 1992; Polich 2007; Brouwer et al. 2012; Zahedi et al. 2020). P3 visual ERP responses have been reported with the Oculus VR system in a bimodal oddball task (Harjunen et al. 2017), a decision-making game paradigm (Spapé et al. 2019), a brain–computer interface (BCI) speller (Du et al. 2019) and in a BCI training paradigm (Amaral et al. 2018). There are only a few investigations which have reported and analysed the early post-stimulus positive and negative ERP components in response to working memory tasks. For example, early responses at ~ 40–150 ms post-stimulus, termed P1 and N1 components, have been reported (Pratt et al. 2011; Liu et al. 2017; Zhao et al. 2020). The N1 and P1 components are thought to reflect early attentional processing and low level features (Hillyard and Anllo-Vento 1998; Woodman 2010; Pratt et al. 2011; Hinojosa et al. 2015; Shalchy et al. 2020).

The use of ERPs to examine the influence of VR HMD or desktop display on measures of cognitive workload may offer potential insights into the underlying cognitive components affecting memory workload. Although the investigations by Dey et al. (2019) and Tremmel et al. (2019) have reported the responses to the *n*-back task using a combination of VR HMD and EEG they undertook only a power spectral analysis of the data, which enables a combined non-time locked and time-locked EEG data analysis of the presented *n*-back stimuli. As far as we are aware, there are no reported studies which have presented an analysis of ERP responses (stimuli time-locked) to a classic *n*-back working memory task using a combined VR HMD with EEG approach and compared the ERP waveforms to those obtained using a desktop CS. Since the visual surround in VR HMD is relatively more controlled compared to the desktop CS, differences may be expected in the N1/P1/P3 ERP responses between the two environments for the presented working memory task (there was no a priori hypothesis on the direction of change). The aim of our comparative electrophysiological brain study is to determine the similarities and differences between VR HMD (without any physical modification or customisation to the headset) and the traditional desktop CS environment in response to a classic visual *n*-back working memory task. Specifically, we compared N1/P1/P3 ERP waveforms using a within group design, where the VR HMD and the control desktop CS protocols were undertaken in the same experimental session day with participants wearing the same EEG electrode headcap.

## Materials and methods

### Participants

**Fig. 1:**
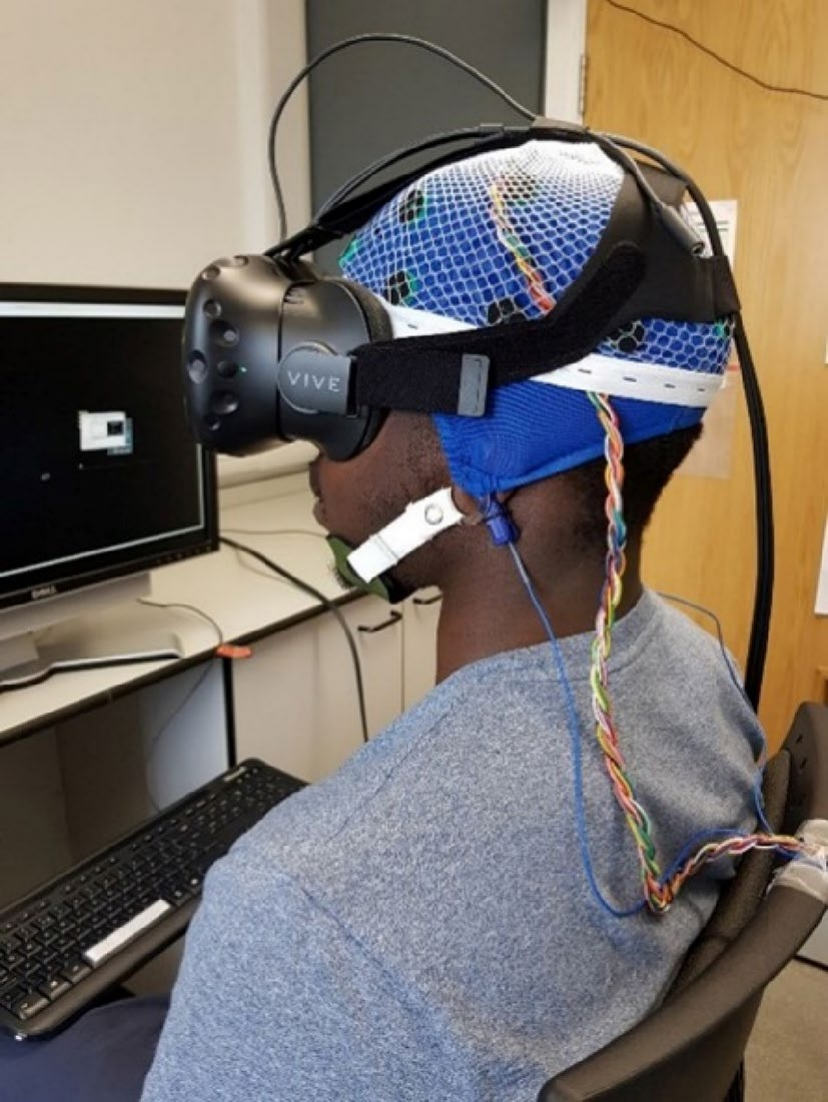
Participant wearing the EEG headcap (blue colour), EEG net (white colour) and the VR HMD system

In previous EEG/ERP studies, authors have typically utilised a range of 10–30 (average ~ 20) participants in their investigations (see Larson and Carbine 2017). In our study, we recruited 21 participants who took part in this study voluntarily and received no payment (7 females and 14 males, ages 20–28, Mean = 23.6, SD = 2.1). Recruitment was achieved through word-of-mouth. All participants were right-handed, according to the Edinburgh Handedness Inventory (Oldfield 1971). Each participant was asked to complete the Spielberger State-Trait Anxiety Inventory (Marteau and Bekker 1992) and a paper-based health questionnaire which served to exclude pre-existing conditions or medications that could affect cognitive function. No participants reported neurological disorders or psychological discomfort, and all participants had normal or corrected-to-normal visual acuity. The study was carried out in conformity with the Declaration of Helsinki (World Medical Association 2013). Local ethical approval was given by the Hull York Medical School Ethics Committee. Informed written consent was gained from each participant before partaking in the study. Participants were free to withdraw from the study at any time without having to provide an explanation. Participants were instructed to verbally report any discomfort experienced whilst wearing the HTC Vive VR HMD and the EEG headcap during the experimental session (Fig. 1). No participants reported any discomfort except one participant (out of 21) who experienced motion sickness during the trial period in the VR HMD environment and the session was terminated.

### Desktop CS and VR HMD

Dell UltraSharp 2408WFP 24 inches LCD Monitor was used to present cognitive tasks in the CS environment (resolution: 1920 × 1200; pixel-response rate: 6 ms; contrast ratio: 3000:1 dynamic; visual angle: 9.8° × 9.8°; a distance of ~ 55 cm from the participant’s nasion). An unmodified HTC Vive VR HMD (first version) was used for generating the virtual environment (display: OLED; resolution: 2160 × 1200; refresh rate: 90 Hz; platform: SteamVR, Viveport; field of view: 110°; weight 470 g). The HTC Vive VR HMD was calibrated to the experimental room using two HTC Vive base stations (version 1.0) which track and trace the VR HMD headset and hand controllers.

For the VR HMD headset, the distance between the VR HMD left and right lenses, and the distance between the participant’s face and the VR HMD lenses, were adjusted for each participant to ensure comfortable vision of the VR environment. The *n*-back task was presented in the HTC Vive VR environment via Bigscreen Beta software (version 0.16.1) which enables proportional mirroring of the CS. The Bigscreen software parameters for curvature and brightness were fixed for all participants at 0% and 75%, respectively. The field of view in the VR HMD environment was expanded to the extent that participants could see the faint outline of the screen border at the periphery of their sight with a black void in the periphery. For each participant, we adjusted the parameters for size and distance (depth); the Mean ± SEM for size = 36.1 ± 2.5%, and distance = 37.3 ± 2.1%.

In a separate experimental session (without any participant involvement), we assessed the intensity of light of the presented stimuli, as this could affect the interpretation of data between the two environments, using a probe of a calibrated light meter (RS PRO ILM1335, RS Components). The probe was placed into the cut-out left eye area of a polystyrene mannequin head model, and the VR HMD headset attached. The light meter was used to measure the intensity of light using the lux scale. In our EEG laboratory, with lights off and blinds closed, the light level range was ~ 0.5–10 lx depending upon the location of measurement. For the CS environment, the light level during the presentation of each *n*-back trial (white colour on black background) was 0.62 lx (0.61 lx for the interval screen between each trial), and for the VR HMD headset 0.14 lx (0.13 lx for the interval screen).

### EEG system

The data was recorded using a smartphone-based EEG system (Clewett et al. 2016; Bateson 2018; Bateson and Asghar 2021), with a sampling rate of 250 Hz, and was affixed to the backrest of a chair upon which the participant was sitting. EEG signals were acquired from 19 Ag/AgCl scalp electrodes (Fp1, Fp2, F7, F3, Fz, F4, F8, T3, C3, Cz, C4, T4, T5, P3, Pz, P4, T6, O1, O2) configured in the international 10–20 arrangement using an EEG headcap (Sleepcap, electrodes encased in a soft sponge material, cables located internally, Spes Medica). A support headcap net (Spes Medica) was placed on top of the EEG Sleepcap to fix in position the Sleepcap electrodes. Two linked Ag/AgCl ear electrodes (clipped to the earlobes) were used as the reference electrode in each participant.

### Cognitive task

**Fig. 2:**
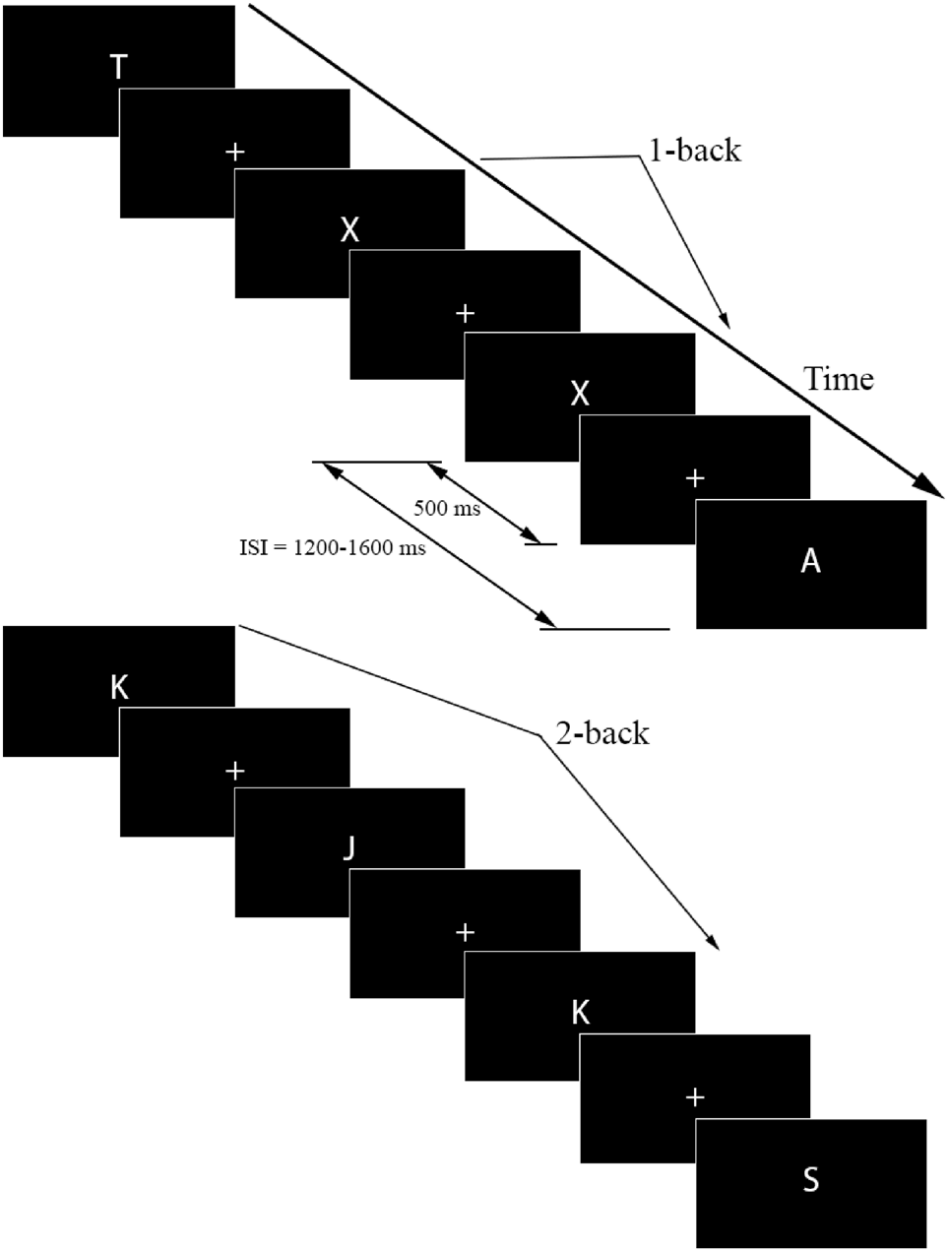
The *n*-back task and illustration of 1-back and 2-back trials, and fixation cross (+). *ISI* Inter-stimulus interval

An *n*-back task was designed and run by employing the Psychology Experiment Building Language (PEBL, version 0.14) open-source software (Mueller and Piper 2014). A single letter appeared in white font on a black background for 500 ms, followed by a fixation cross which was jittered 700–1100 ms (Fig. 2).

The 1-back and 2-back cognitive task conditions were presented to the participants. An initial practice session was undertaken which presented both 1-back and 2-back tasks. The participants received instructions that informed them regarding the particular *n*-back task (1-back or 2-back) to be undertaken in the experimental session. In each session (CS or VR HMD) participants completed the 1-back task before continuing onto the 2-back task as undertaken by Brouwer et al. (2012), Scharinger et al. (2017) and Liu et al. (2017).

The 1-back task comprised 101 trials and the 2-back task comprised 102 trials (English alphabet letters). We sought to balance the requirement for an adequate number of target stimuli for the ERP analysis with the recording session duration in which participants could comfortably complete the experimental protocol in CS and VR HMD environments. To ensure that the ERP protocol was ~ 50 min in duration, we selected 40 target stimuli per condition. A participant break of 10–15 min was provided between the change-over of the screen environments to reduce the potential for fatigue.

The sequence of letter stimuli was pseudo-randomised (to stop repeat of the same letter sequence and to ensure that lure trials were not present) for each of the two conditions. During each block, participants were required to respond by pressing the space bar key on a computer keyboard with their dominant hand when the current trial matched the stimulus seen *n*-back steps ago (1-back or 2-back). To enable participants to feel and easily locate the space bar key whilst wearing the HTC Vive VR HMD this key was fully covered in self-adhesive loop strip tape (VELCRO®). The same keyboard with the loop strip on the spacebar key was used for the *n*-back task presented in the CS environment. Participants were instructed to sit in the same posture for the CS and VR HMD experimental sessions.

### Experimental procedure

The electrophysiological experiments took place in a dimly lit room, at room temperature. Each participant was provided with earplugs and completed the *n*-back task both in VR HMD and CS environments. The order in which the participants completed the *n*-back task was pseudo-randomised (VR first and then CS, or CS first and then VR), and counterbalanced at 50:50. The participant wore the same headcap (without removal) for both the CS and VR experimental sessions, and EEG electrode impedance was checked at the start of each session (each electrode impedance was kept below 5 kΩ).

### EEG data pre-processing

EEG data were processed and analysed using EEGLAB (Delorme and Makeig 2004) and ERPLAB (Lopez-Calderon and Luck 2014) toolboxes running on MATLAB (Matlab R2015a, Mathworks, Inc.). Previous investigations of ERP components during VR HMD have used a range of high pass filters from 0.1 to 3 Hz (Harjunen et al. 2017; Rupp et al. 2019; Tauscher et al. 2019; Du et al. 2019; Lier et al. 2020). In our study, we applied a 2nd order infinite impulse response (IIR) Butterworth filter for bandpass filtering to the continuous data with a lower cutoff of 0.5 Hz and higher cutoff of 30 Hz. All data sets were segmented into epochs from − 200 to 600 ms relative to the stimulus onset. A 200 ms pre-stimulus time window was used for baseline correction.

Artifact detection was applied to the epoched data (target and non-target trials) by employing a moving window peak-to-peak function with a 100 µV voltage threshold (Luck 2014). Next, a visual inspection was performed to detect and remove epochs with residual artifacts. We selected trials for inclusion in which participant’s selected correct responses to the target trials. Previous investigations have indicated that a minimum of 6–8 trials are necessary for accurate and reasonably stable ERP analysis (Olvet and Hajcak 2009; Pontifex et al. 2010; Boudewyn et al. 2018). Across the participants, the mean ± SEM and range of correct target stimuli epochs selected for the data analysis for CS and VR HMD environments were 17 ± 1.47 (range 10–34) and 21 ± 1.49 (range 11–35), respectively. Target ERP waveforms obtained from each participant were averaged for each environment across the 1-back and 2-back conditions.

### Measurement of ERPs

**Fig. 3:**
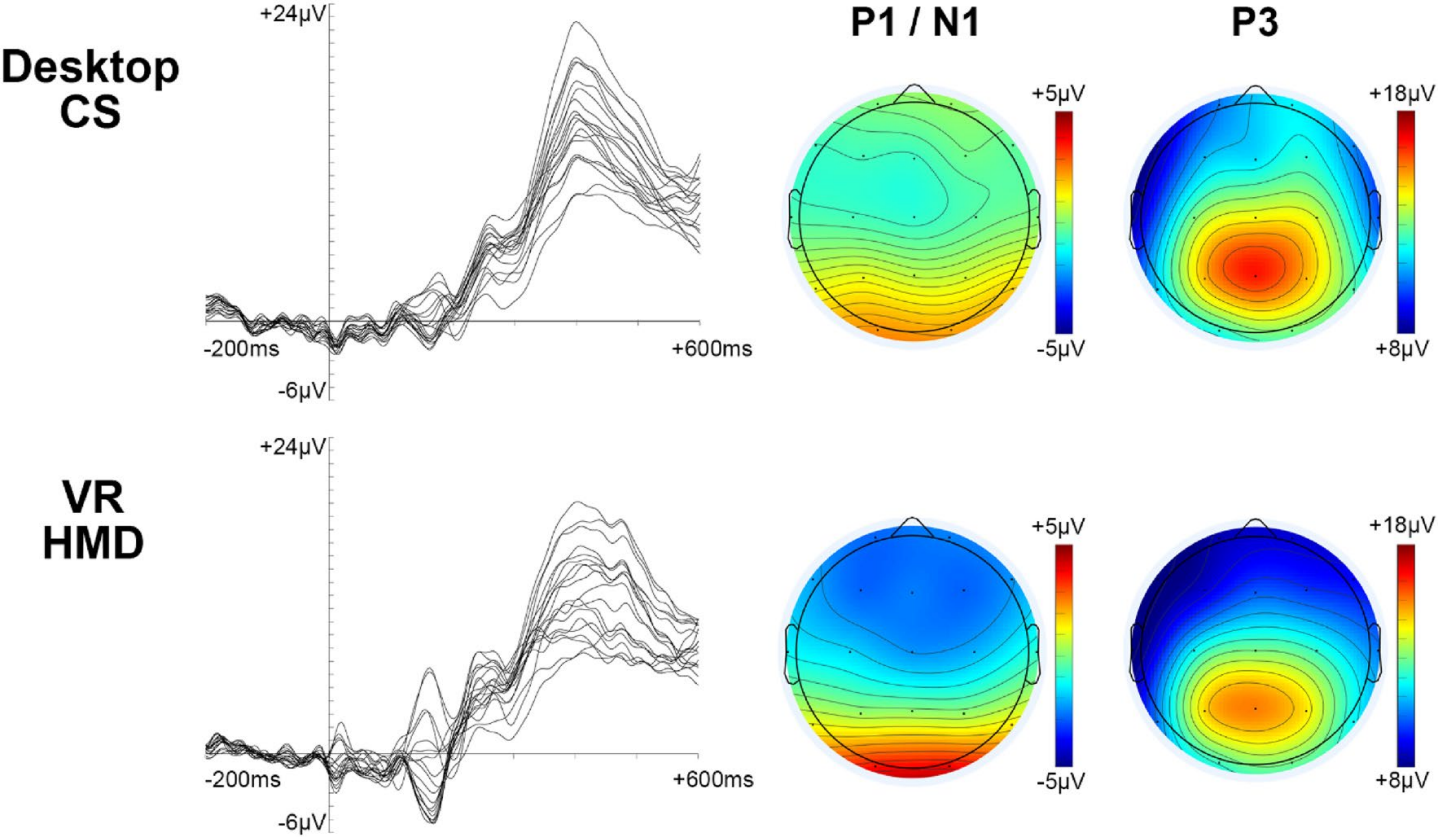
ERP responses to all 19 EEG electrode locations (each channel averaged across 20 participants) in Desktop CS and VR HMD environments (combined 1-back and 2-back conditions) and voltage topography maps for the P1/N1 (120–200 ms) and P3 (300–500 ms) components

The ERP responses acquired in both CS and VR HMD environments were visually inspected for components and their durations (Fig. 3). Based upon this visualisation, and previous ERP investigations using working memory tasks (Pratt et al. 2011; Liu et al. 2017; Zhao et al. 2020), the anterior N1, posterior P1 and P3 components were selected for the ERP analysis. The first negative waveform that peaked between 120 and 200 ms after stimulus onset at the frontal and central regions was identified and named as N1. The first positive peak within 120–200 ms after stimulus onset at the temporal and occipital regions was named as P1. A positive amplitude waveform, observed between 300 and 500 ms after stimulus onset, was labelled as P3.

The peak amplitude (local peak approach) and peak latency are commonly used metrics which investigators measure in their analysis of a given ERP component (Luck 2014). Peak amplitude and latency of the ERP component at a single timepoint have been measured in *n*-back studies (Watter et al. 2001; Hajcak et al. 2010; Daffner et al. 2011; Brouwer et al. 2012; Chen et al. 2019) including an investigation using VR HMD (Harjunen et al. 2017). An alternative to using peak metrics, which is increasingly utilised, is the mean amplitude measure (Luck 2014; Nielsen and Gonzalez 2020). The mean amplitude measure has the advantage of taking into account the selected ERP component over a given time period instead of at a single timepoint only, although there could be shortcomings in terms of time window selection as narrower windows may increase the noise level (Luck 2014; Hutman et al. 2016; Canette et al. 2020). In our ERP analysis, we have utilised both a peak and mean amplitude measures approach to take advantage of both metrics as has been undertaken by other investigators (Luck 2014; Hutman et al. 2016; Canette et al. 2020).

Mean amplitude (integral/time period selected, μV), peak amplitude (μV) and peak latency (ms) measurements of N1, P1 and P3 components were calculated for the following electrode clusters: N1: frontal (F3 + Fz + F4) and central (C3 + Cz + C4); P1: temporal (T5 + T6) and occipital (O1 + O2); P3: frontal (F3 + Fz + F4), central (C3 + Cz + C4), parietal (P3 + Pz + P4), temporal (T5 + T6) and occipital (O1 + O2). This combination of electrodes selected at each region was based upon those utilised in previously published ERP investigations (see Scharinger et al. 2017; Watter et al. 2001).

### Statistical analysis of behavioural and electrophysiological data

For the behavioural analyses, response times (correct responses) and accuracy (% of correct responses) of the participants were calculated for each participant separately. Behavioural data (response time or accuracy) were analysed using a 2 × 2 [environment (CS, VR HMD) × cognitive workload (1-back, 2-back)] repeated-measures analysis of variance (ANOVA).

The ERP data obtained for the N1 component was analysed using a 2 × 2 × 2 [environment (CS, VR HMD) × cognitive workload (1-back, 2-back) × region (frontal, central)] repeated-measures ANOVA separately for each dependent variable (mean amplitude, peak amplitude, peak latency). Likewise, the P1 was analysed with a 2 × 2 × 2 [environment (CS, VR HMD) × cognitive workload (1-back, 2-back) × region (temporal, occipital)] repeated-measures ANOVA. Finally, the P3 component was analysed using a 2 × 2 × 5 [environment (CS, VR HMD) × cognitive workload (1-back, 2-back) × region (frontal, central, parietal, temporal, occipital)] ANOVA.

The statistical analysis of the data was performed using IBM SPSS Statistics software (version 24, SPSS Inc., Chicago, IL, USA). Bonferroni adjustment (*p* < 0.05) was used for the post-hoc pairwise comparisons and paired-samples *t* tests. The Greenhouse–Geisser epsilon correction was applied when the sphericity assumption was violated (Mauchly’s test of sphericity).

## Results

### Behavioural results

Behavioural results of mean correct response rates (accuracy) and reaction times of participants are presented in Table 1. For the accuracy of correct responses, a two-way repeated-measures ANOVA was conducted and a significant main effect found for cognitive workload *F*(1, 19) = 45.425, *p* < 0.001, *η*_p_^2^ = 0.71 but not for environment *F*(1, 19) = 1.902, *p* = 0.184, *η*_p_^2^ = 0.09 or the interaction effect between environment and cognitive workload *F*(1, 19) = 0.339, *p* = 0.567, *η*_p_^2^ = 0.02. Post-hoc pairwise *t* tests with Bonferroni adjustment showed that the accuracy differed significantly between 1-back and 2-back levels of cognitive workload. The accuracy level, irrespective of environment, in the 1-back condition [Mean ± SEM (*M*) = 89.00 ± 0.03] was significantly higher compared to the 2-back [*M* = 69.00 ± 0.04, *t*(19) = 6.71, *p* < 0.001, *d* = 1.50].

**Table 1:** Mean correct response rates (accuracy) and reaction times for participants undertaking the *n*-back task in CS and VR HMD environments

	1-back		2-back	
	CS	VR HMD	CS	VR HMD
Mean correct response rate (%)	86.9 ± 4.2	91.1 ± 2.9	68.0 ± 4.1	69.9 ± 3.7
Mean reaction time (ms)	504.5 ± 19.6	487.9 ± 16.6	550.1 ± 18.2	548.0 ± 18.7

Values (*n* = 20) are mean ± SEM

For the mean reaction times, the results of the two-way repeated-measures ANOVA indicated that there was a significant main effect of cognitive workload *F*(1, 19) = 12.295, *p* = 0.002, *η*_p_^2^ = 0.40, during the *n*-back tasks. Pairwise comparison with Bonferroni adjustment revealed that there was a significant increase in reaction times from 1-back (*M* = 496.17 ± 17.10) to 2-back (*M* = 549.11 ± 16.44) irrespective of environment [*t*(19) = − 3.51, *p* = 0.002, *d* = − 0.78]. There was no main effect of environment *F*(1, 19) = 0.573, *p* = 0.458, *η*_p_^2^ = 0.03 or an interaction effect between environment and cognitive workload *F*(1, 19) = 0.577, *p* = 0.457, *η*_p_^2^ = 0.03.

### ERP results

**Fig. 4:**
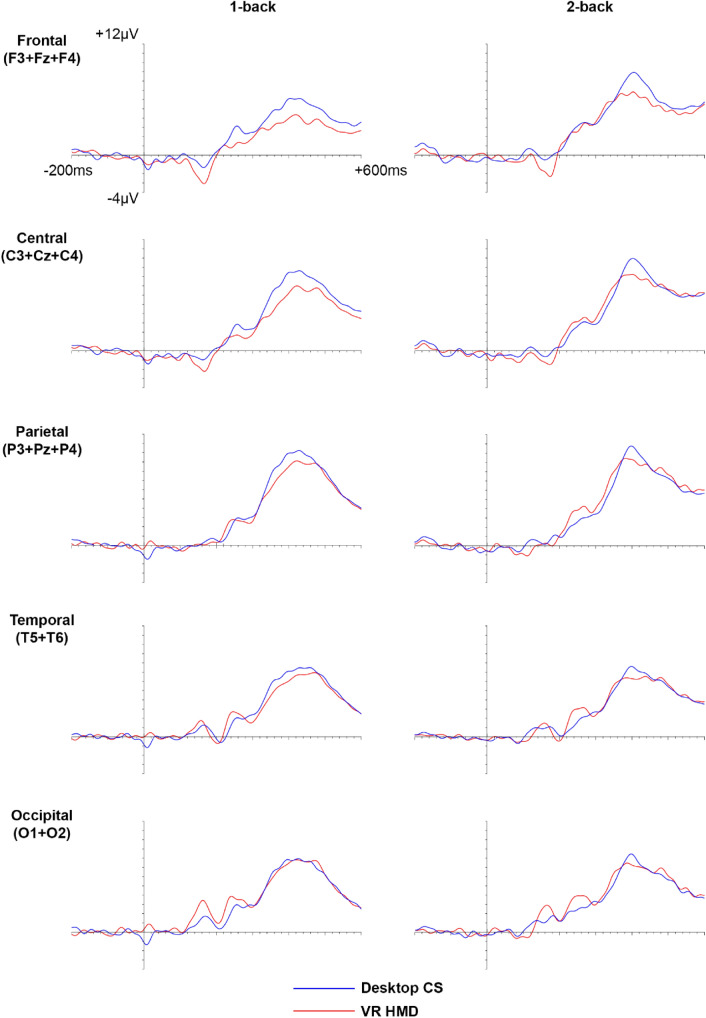
Grand average ERP responses to both 1-back and 2-back conditions in the CS and VR HMD environments at frontal (F3 + Fz + F4), central (C3 + Cz + C4), parietal (P3 + Pz + P4), temporal (T5 + T6) and occipital (O1 + O2) regions

The grand average ERP responses to both 1-back and 2-back conditions in the CS and VR HMD environments are presented in Fig. 4. We measured the mean amplitude, peak amplitude and latency of the N1, P1 and P3 ERP components in response to the presentation of the 1-back and 2-back tasks.

#### N1 component

In the frontal (F3 + Fz + F4) and central (C3 + Cz + C4) regions an N1 component (between 120 and 200 ms) was observed. Measurements for mean amplitude, peak amplitude and peak latency of N1 for CS or VR HMD, with 1-back and 2-back workloads in frontal and central regions, are presented in Table 2. All the statistical analyses are summarised in Table 3.

**Table 2:** Mean amplitude, peak amplitude and latency for N1 and P1 components in CS and VR HMD environments at 1-back and 2-back levels of cognitive workload

	1-back		2-back	
	CS	VR HMD	CS	VR HMD
N1				
Mean amplitude (μV)				
Frontal	− 0.6 ± 0.4	− 1.7 ± 0.5	0.1 ± 0.4	− 1.1 ± 0.4
Central	− 0.6 ± 0.4	− 1.2 ± 0.4	− 0.2 ± 0.4	− 0.8 ± 0.4
Peak amplitude (μV)				
Frontal	− 2.2 ± 0.5	− 3.7 ± 0.6	− 2.0 ± 0.5	− 3.6 ± 0.5
Central	− 2.0 ± 0.4	− 2.9 ± 0.5	− 2.1 ± 0.4	− 2.9 ± 0.5
Peak latency (ms)				
Frontal	160.8 ± 3.8	161.2 ± 2.9	164.7 ± 5.0	163.3 ± 2.8
Central	162.3 ± 4.1	158.8 ± 3.4	163.9 ± 3.8	158.5 ± 4.1
P1				
Mean amplitude (μV)				
Temporal	0.5 ± 0.4	0.7 ± 0.5	0.6 ± 0.4	0.5 ± 0.5
Occipital	0.9 ± 0.5	2.1 ± 0.7	1.0 ± 0.5	1.7 ± 0.8
Peak amplitude (μV)				
Temporal	2.2 ± 0.4	2.6 ± 0.5	2.6 ± 0.5	2.9 ± 0.5
Occipital	2.8 ± 0.6	4.2 ± 0.9	3.4 ± 0.6	4.3 ± 1.0
Peak latency (ms)				
Temporal	164.2 ± 2.9	156.0 ± 3.4	162.7 ± 3.5	157.8 ± 4.2
Occipital	168.9 ± 4.3	164.4 ± 2.4	165.6 ± 5.0	166.6 ± 3.5

Values (*n* = 20) are mean ± SEM

**Table 3:** ERP ANOVA results for the N1 component at the frontal and central regions

Effect (N1)	*F*	*p*	*η*_p_^2^
Mean amplitude			
Environment	4.142	0.056	0.18
Cognitive workload	3.124	0.093	0.14
Region	0.535	0.473	0.03
Environment × Cognitive workload	0.021	0.887	< 0.01
Environment × Region	**14.187**	0.001**	**0.43**
Cognitive workload × Region	1.077	0.312	0.05
Environment × Cognitive workload × Region	0.122	0.730	0.01
Peak amplitude			
Environment	**6.020**	0.024*	**0.24**
Cognitive Workload	0.002	0.967	< 0.01
Region	**4.993**	0.038*	**0.21**
Environment × Cognitive workload	0.001	0.977	< 0.01
Environment × Region	**14.010**	0.001**	**0.42**
Cognitive workload × Region	0.859	0.366	0.04
Environment × Cognitive workload × Region	0.209	0.653	0.01
Peak latency			
Environment	0.455	0.508	0.02
Cognitive workload	0.412	0.529	0.02
Region	1.146	0.298	0.06
Environment × Cognitive workload	0.129	0.723	0.01
Environment × Region	1.542	0.229	0.08
Cognitive workload × Region	0.768	0.392	0.04
Environment × Cognitive workload × Region	< 0.001	0.991	< 0.01

Bold print and *p < 0.05 and **p < 0.01 indicate a statistically significant difference

#### N1 mean amplitude

The results of the three-way repeated-measures ANOVA showed no significant main effects of environment *F*(1, 19) = 4.142, *p* = 0.056, *η*_p_^2^ = 0.18, cognitive workload *F*(1, 19) = 3.124, *p* = 0.093, *η*_p_^2^ = 0.14 and region *F*(1, 19) = 0.535, *p* = 0.473, *η*_p_^2^ = 0.03 on the N1 mean amplitude at the frontal and central regions.

There was a significant interaction effect between environment and region *F*(1, 19) = 14.187, *p* = 0.001, *η*_p_^2^ = 0.43. A subsequent paired *t* test revealed that the N1 mean amplitude measured in the VR HMD environment (*M* = − 1.40 ± 0.39) was significantly higher than the CS environment (*M* = − 0.23 ± 0.34) at the frontal region [*t*(19) = 2.49, *p* = 0.044, *d* = 0.56]. There was no significant difference between the VR HMD (*M* = − 1.01 ± 0.35) and CS (*M* = − 0.40 ± 0.30) environments [*t*(19) = 1.47, *p* = 0.318, *d* = 0.33] for the N1 mean amplitude measured at the central region. There were no other interaction effects on the N1 mean amplitude (all values of *p* > 0.05).

#### N1 peak amplitude

The three-way repeated-measures ANOVA revealed a significant main effect of environment *F*(1, 19) = 6.020, *p* = 0.024, *η*_p_^2^ = 0.24. Post-hoc pairwise comparisons showed that the N1 peak amplitudes for the VR HMD environment (*M* = − 3.27 ± 0.46) were significantly higher compared to CS environment [*M* = − 2.05 ± 0.35, *t*(19) = 2.46, *p* = 0.024, *d* = 0.55]. There was also a significant main effect of region *F*(1, 19) = 4.993, *p* = 0.038, *η*_p_^2^ = 0.21, reflecting a significant increase in the N1 peak amplitude at the frontal region (*M* = − 2.87 ± 0.36) compared to the central region [*M* = − 2.46 ± 0.31, *t*(19) = − 2.24, *p* = 0.038, *d* = − 0.50]. There was no significant main effect of cognitive workload *F*(1, 19) = 0.002, *p* = 0.967, *η*_p_^2^ < 0.01 on the N1 peak amplitude at frontal and central regions.

A significant interaction effect was found between environment and region *F*(1, 19) = 14.010, *p* = 0.001, *η*_p_^2^ = 0.42. There were no other significant interaction effects between the factors (all values of *p* > 0.05). A post-hoc paired *t* test was conducted to examine this interaction effect. This analysis revealed a significant increase in the N1 peak amplitude at the frontal region for the VR environment (*M* = − 3.67 ± 0.49) compared to the CS environment [*M* = − 2.07 ± 0.40, *t*(19) = 2.97, *p* = 0.016, *d* = 0.66]. There was no significant difference between the VR (*M* = − 2.88 ± 0.43) and CS (*M* = − 2.04 ± 0.34) environments at the central region [*t*(19) = 1.77, *p* = 0.184, *d* = 0.40].

#### N1 peak latency

The three-way repeated-measures ANOVA showed no significant effects of any factors or their interactions (all values of *p* > 0.05) for the N1 peak latency at the frontal and central regions.

#### P1 component

Mean amplitude, peak amplitude, and peak latency of the P1 component (between 120 and 200 ms) were calculated at the temporal (T5 + T6) and occipital (O1 + O2) regions (Table 2). All the statistical analyses are summarised in Table 4.

**Table 4:** ERP ANOVA results for the P1 component at the temporal and occipital regions

Effect (P1)	*F*	*p*	*η*_p_^2^
Mean amplitude			
Environment	2.088	0.165	0.10
Cognitive workload	0.148	0.705	0.01
Region	**8.799**	0.008**	**0.32**
Environment × Cognitive workload	0.592	0.451	0.03
Environment × Region	**8.978**	0.007**	**0.32**
Cognitive workload × Region	0.559	0.464	0.03
Environment × Cognitive workload × Region	1.968	0.177	0.09
Peak amplitude			
Environment	3.064	0.096	0.14
Cognitive workload	1.968	0.177	0.09
Region	**6.813**	0.017*	**0.26**
Environment × Cognitive workload	0.198	0.661	0.01
Environment × Region	2.898	0.105	0.13
Cognitive workload × Region	0.017	0.869	< 0.01
Environment × Cognitive workload × Region	1.660	0.213	0.08
Peak latency			
Environment	1.945	0.179	0.09
Cognitive workload	0.008	0.928	< 0.01
Region	**5.636**	0.028*	**0.23**
Environment × Cognitive workload	0.638	0.434	0.03
Environment × Region	1.883	0.186	0.09
Cognitive workload × Region	0.052	0.822	< 0.01
Environment × Cognitive workload × Region	0.250	0.623	0.01

Bold print and *p < 0.05 and **p < 0.01 indicate a statistically significant difference

#### P1 mean amplitude

For the P1 mean amplitude, there was a significant main effect of region *F*(1, 19) = 8.799, *p* = 0.008, *η*_p_^2^ = 0.32, where there was a significant increase in the P1 mean amplitude at the occipital region (*M* = 1.42 ± 0.56) compared to the temporal region [*M* = 0.58 ± 0.37, *t*(19) = − 2.97, *p* = 0.008, *d* = − 0.66]. There were no significant main effects of environment *F*(1, 19) = 2.088, *p* = 0.165, *η*_p_^2^ = 0.10 and cognitive workload *F*(1, 19) = 0.148, *p* = 0.705, *η*_p_^2^ = 0.01 on the P1 mean amplitude.

A significant interaction effect was found between environment and region *F*(1, 19) = 8.978, *p* = 0.007, *η*_p_^2^ = 0.32. Post-hoc comparisons showed a significantly higher P1 mean amplitude in the VR HMD environment for the occipital region (*M* = 1.90 ± 0.72) compared to the temporal [*M* = 0.61 ± 0.45, *t*(19) = − 3.41, *p* < 0.001, *d* = − 1.09]. No significant difference was found between the temporal (*M* = 0.54 ± 0.34) and occipital [*M* = 0.94 ± 0.46, *t*(19) = − 1.60 *p* = 0.508, *d* = − 0.40] regions for the P1 mean amplitude in the CS environment. There were no significant differences in the P1 mean amplitude responses between VR HMD (temporal: *M* = 0.61 ± 0.45, occipital: *M* = 1.90 ± 0.72) and CS (temporal: *M* = 0.54 ± 0.34, occipital: *M* = 0.94 ± 0.46) environments at both temporal [*t*(19) = − 0.262, *p* = 1.000, *d* = − 0.06] and occipital [*t*(19) = − 2.05, *p* = 0.216, *d* = − 0.46] regions during the *n*-back tasks. There were no other interaction effects on the P1 mean amplitude (all values of *p* > 0.05).

#### P1 peak amplitude

A three-way repeated-measures ANOVA was conducted to examine the effects of environment (CS, VR HMD), cognitive workload (1-back, 2-back) and region (temporal, occipital). The results showed only a significant main effect for region *F*(1, 19) = 6.813, *p* = 0.017, *η*_p_^2^ = 0.26 for the P1 peak amplitude. A post-hoc test with Bonferroni correction showed that the P1 peak amplitude at the occipital region (*M* = 3.67 ± 0.68) was significantly higher than at the temporal region [*M* = 2.56 ± 0.40, *t*(19) = − 2.61, *p* = 0.017, *d* = − 0.58]. There were no significant main effects of environment *F*(1, 19) = 3.064, *p* = 0.096, *η*_p_^2^ = 0.14 and cognitive workload *F*(1, 19) = 1.968, *p* = 0.177, *η*_p_^2^ = 0.09 as well as no interaction effects between the factors (all values of *p* > 0.05).

#### P1 peak latency

A three-way repeated-measures ANOVA was performed for the P1 peak latency at the temporal–occipital regions during the *n*-back tasks. There was a significant main effect of region *F*(1, 19) = 5.636, *p* = 0.028, *η*_p_^2^ = 0.23 on the P1 peak latency, reflecting earlier P1 peaks at the temporal region (*M* = 160.18 ± 2.57) compared to the occipital region [*M* = 166.38 ± 2.67, *t*(19) = − 2.37, *p* = 0.028, *d* = − 0.53]. No main effects were found for environment *F*(1, 19) = 1.945, *p* = 0.179, *η*_p_^2^ = 0.09 and cognitive workload *F*(1, 19) = 0.008, *p* = 0.928, *η*_p_^2^ = 0.00 or interaction effects (all values of *p* > 0.05).

#### P3 component

The P3 component (between 300 and 500 ms) was measured at the frontal (F3 + Fz + F4), central (C3 + Cz + C4), parietal (P3 + Pz + P4), temporal (T5 + T6) and occipital (O1 + O2) electrode cluster locations (Table 5). The results of the statistical analyses are presented in Table 6.

**Table 5:** Mean amplitude, peak amplitude and latency for the P3 component in CS and VR HMD environments at 1-back and 2-back levels of cognitive workload

P3	1-back		2-back	
	CS	VR HMD	CS	VR HMD
Mean amplitude (μV)				
Frontal	4.7 ± 0.8	3.5 ± 0.9	6.8 ± 1.0	5.8 ± 0.9
Central	6.6 ± 1.0	5.5 ± 1.1	7.5 ± 1.1	7.3 ± 0.9
Parietal	8.1 ± 1.1	7.5 ± 1.2	8.0 ± 1.1	8.4 ± 1.0
Temporal	6.0 ± 0.8	5.4 ± 0.8	5.8 ± 0.8	5.7 ± 0.8
Occipital	6.5 ± 0.9	6.6 ± 1.1	6.2 ± 1.0	6.6 ± 1.0
Peak amplitude (μV)				
Frontal	8.1 ± 1.0	6.2 ± 1.0	10.3 ± 1.0	9.2 ± 1.0
Central	10.3 ± 1.4	8.8 ± 1.2	11.7 ± 1.2	11.0 ± 1.1
Parietal	12.1 ± 1.4	11.2 ± 1.3	12.4 ± 1.3	12.3 ± 1.2
Temporal	9.1 ± 1.0	8.6 ± 0.8	9.2 ± 1.0	9.0 ± 0.8
Occipital	10.0 ± 1.1	9.9 ± 1.1	10.1 ± 1.1	9.9 ± 1.1
Peak latency (ms)				
Frontal	427.2 ± 10.5	405.7 ± 11.7	411.7 ± 8.0	408.5 ± 11.3
Central	432.8 ± 10.1	424.6 ± 9.4	416.2 ± 7.5	421.0 ± 9.8
Parietal	428.7 ± 9.3	429.5 ± 8.6	415.1 ± 8.0	418.5 ± 10.6
Temporal	436.8 ± 8.9	432.6 ± 9.5	419.3 ± 9.0	423.9 ± 10.6
Occipital	428.4 ± 10.1	423.9 ± 10.3	411.3 ± 9.2	412.0 ± 10.1

Values (*n* = 20) are mean ± SEM

**Table 6:** ERP ANOVA results for the P3 component at the frontal, central, parietal, temporal and occipital regions

Effect (P3)	*F*	*p*	*η*_p_^2^
Mean amplitude			
Environment	0.869	0.363	0.04
Cognitive workload	2.672	0.119	0.12
Region	**6.332**	0.005**	**0.25**
Environment × Cognitive workload	0.362	0.555	0.02
Environment × Region	2.502	0.094	0.12
Cognitive workload × Region	**13.154**	< 0.001**	**0.41**
Environment × Cognitive workload × Region	0.693	0.534	0.04
Peak amplitude			
Environment	2.321	0.144	0.11
Cognitive workload	3.229	0.088	0.15
Region	**8.368**	0.001**	**0.31**
Environment × Cognitive workload	0.193	0.666	0.01
Environment × Region	2.617	0.080	0.12
Cognitive workload × Region	**10.867**	< 0.001**	**0.36**
Environment × Cognitive workload × Region	0.448	0.693	0.02
Peak latency			
Environment	0.275	0.606	0.01
Cognitive workload	**5.175**	0.035*	**0.21**
Region	**3.841**	0.024*	**0.17**
Environment × Cognitive workload	0.684	0.418	0.04
Environment × Region	1.082	0.362	0.05
Cognitive workload × Region	0.375	0.826	0.02
Environment × Cognitive workload × Region	0.333	0.741	0.02

Bold print and *p < 0.05 and **p < 0.01 indicate a statistically significant difference

#### P3 mean amplitude

Using a three-way repeated-measures ANOVA for the mean P3 amplitude, a significant main effect was seen for region *F*(1.88, 35.76) = 6.332, *p* = 0.005, ε = 0.47, *η*_p_^2^ = 0.25, showing a significantly higher mean P3 amplitude at the parietal region (*M* = 8.00 ± 0.98) compared to central [*M* = 6.73 ± 0.90, *t*(19) = 3.51, *p* = 0.024, *d* = 0.78], occipital [*M* = 6.47 ± 0.90, *t*(19) = 3.19, *p* = 0.048, *d* = 0.71], temporal [*M* = 5.73 ± 0.70, *t*(19) = 4.88, *p* = 0.001, *d* = 1.10)] and frontal [*M* = 5.21 ± 0.74, *t*(19) = 4.00, *p* = 0.008, *d* = − 0.89)] regions. There was no main effect for environment *F*(1, 19) = 0.869, *p* = 0.363, *η*_p_^2^ = 0.04 or cognitive workload *F*(1, 19) = 2.672, *p* = 0.119, *η*_p_^2^ = 0.12.

A significant interaction effect was only observed between cognitive workload and region *F*(2.01, 38.17) = 13.154, *p* < 0.001, ε = 0.50, *η*_p_^2^ = 0.41. A paired-samples *t* test showed a significant difference in the P3 mean amplitudes between the 1-back (*M* = 4.14 ± 0.74) and 2-back (*M* = 6.29 ± 0.86) conditions at the frontal region [*t*(19) = − 3.433, *p* = 0.015, *d* = − 0.77]. However, there was no significant difference between 1-back and 2-back conditions at the central [1-back: *M* = 6.06 ± 0.97, 2-back: *M* = 7.39 ± 0.92, *t*(19) = − 2.376, *p* = 0.140, *d* = − 0.53], parietal [1-back: *M* = 7.80 ± 1.06, 2-back: *M* = 8.19 ± 0.96, *t*(19) = − 0.736, *p* = 1.000, *d* = − 0.16], temporal [1-back: *M* = 5.69 ± 0.74, 2-back: *M* = 5.76 ± 0.72, *t*(19) = − 0.155, *p* = 1.000, *d* = − 0.03] and occipital [1-back: *M* = 6.53 ± 0.95, 2-back: *M* = 6.41 ± 0.90, *t*(19) = 0.283, *p* = 1.000, *d* = 0.06] regions.

#### P3 peak amplitude

A three-way repeated measures ANOVA revealed that there was a significant main effect of region *F*(1.96, 37.18) = 8.368, *p* = 0.001, ε = 0.49, *η*_p_^2^ = 0.31. Post-hoc pairwise comparisons, using a Bonferroni correction, showed a significantly higher P3 amplitude for the parietal region (*M* = 11.99 ± 1.13) compared to central [*M* = 10.44 ± 1.05, *t*(19) = 3.46, *p* = 0.012, *d* = 0.77], occipital [*M* = 9.97 ± 1.00, *t*(19) = 3.65, *p* = 0.008, *d* = 0.82], temporal [*M* = 8.98 ± 0.76, *t*(19) = 5.34, *p* < 0.001, *d* = 1.19] and frontal [*M* = 8.46 ± 0.81, *t*(19) = 4.55, *p* < 0.001, *d* = 1.02] regions. There was no main effect for environment *F*(1, 19) = 2.321, *p* = 0.144, *η*_p_^2^ = 0.11 or cognitive workload *F*(1, 19) = 3.229, *p* = 0.088, *η*_p_^2^ = 0.15 on the P3 peak amplitude.

There was a significant interaction effect between cognitive workload and region *F*(1.86, 35.62) = 10.867, *p* < 0.001, ε = 0.47, *η*_p_^2^ = 0.36. All other interaction effects were not significant (all values of *p* > 0.05). Using a post-hoc paired *t* test, a significant difference was found in the P3 peak amplitudes between 1-back (*M* = 7.16 ± 0.85) and 2-back (*M* = 9.77 ± 0.94) conditions at the frontal region [*t*(19) = − 3.463, *p* = 0.015, *d* = − 0.77]. However, there was no significant difference between 1-back and 2-back conditions at the central [1-back: *M* = 9.55 ± 1.18, 2-back: *M* = 11.33 ± 1.04, *t*(19) = − 2.458, *p* = 0.120, *d* = − 0.55], parietal [1-back: *M* = 11.64 ± 1.25, 2-back: *M* = 12.35 ± 1.12, *t*(19) = − 1.017, *p* = 1.000, *d* = − 0.23], temporal [1-back: *M* = 8.86 ± 0.82, 2-back: *M* = 9.08, ± 0.79, *t*(19) = − 0.394, *p* = 1.000, *d* = − 0.09] and occipital [1-back: *M* = 9.94 ± 1.06, 2-back: *M* = 10.00 ± 1.02, *t*(19) = − 0.105, *p* = 1.000, *d* = − 0.02] regions.

#### P3 peak latency

The results of the three-way repeated-measures ANOVA for the P3 peak latencies revealed that there were significant main effects of cognitive workload *F*(1, 19) = 5.175, *p* = 0.035, *η*_p_^2^ = 0.21 and of region *F*(2.31, 43.79) = 3.841, *p* = 0.024, ε = 0.58, *η*_p_^2^ = 0.17 but no main effect of environment *F*(1, 19) = 0.275, *p* = 0.606, *η*_p_^2^ = 0.01. The post-hoc results showed that the P3 component peaked significantly earlier for the 2-back (*M* = 415.76 ± 7.60) than the 1-back condition [*M* = 427.02 ± 7.30, *t*(19) = 2.28, *p* = 0.035, *d* = 0.51]. There were no significant interaction effects between the factors (all values of *p* > 0.05).

## Discussion

In our experimental investigation, we successfully acquired ERPs waveforms in response to a visual *n*-back cognitive task in participants wearing an unmodified HTC Vive VR HMD system on top of the EEG headcap. We used a within group design, where the VR HMD and the control desktop CS experimental protocols were held in the same session with participants wearing the same electrode headcap. In our CS control environment, the ERP responses to the *n*-back task contained the P3, N1 and P1 waveform components of interest. The peak amplitudes and latencies of these components were similar to those shown and reported previously in ERP studies of *n*-back using traditional CS setups (Brouwer et al. 2012; Chen et al. 2019; Daffner et al. 2011; Hajcak et al. 2010; Kwon et al. 2018; Scharinger et al. 2015; Watter et al. 2001). In our investigation, we found that the P3, N1 and P1 waveform components followed a similar time course in both VR HMD and CS environments at all EEG electrode locations.

The most prominent amplitude ERP waveform feature we observed in response to the *n*-back task for both CS and VR environments was the P3 component. No main effect of environment was found for this component (mean/peak amplitudes or peak latency), indicating the similarity of the P3 waveforms obtained in VR HMD and CS environments. Our results revealed that the mean and peak P3 amplitudes were significantly higher in the parietal region for both VR and CS environments compared to the other brain regions. Similarly, (Spapé et al. 2019) found the highest P3 amplitude at the parietal Pz electrode using the Oculus Rift VR HMD system with a decision-making game task. In addition, the comparative experimental study of Harjunen et al. (2017) also revealed the highest P3 peak amplitude to a cross-modal paradigm at the parietal Pz electrode using either Oculus Rift VR HMD or desktop CS environments. Our results and those of Harjunen et al. (2017) show that the higher amplitudes at the parietal region seen for the P3 component in the CS environment remains high when the task is also presented in the VR HMD environment. Our ERP results indicate that usage of the VR HMD does not impact upon the P3 waveform and is similar to that obtained in the CS environment.

In our study, a main effect of environment was found for the N1 peak amplitude, where it was significantly higher in the VR HMD environment compared to CS. Furthermore, a significant interaction effect between environment and region revealed a higher N1 peak amplitude at the frontal region compared to the central one. For the P1 mean amplitude, a significant interaction effect between environment and region was also found, showing that there was a higher P1 mean amplitude for VR HMD compared to CS at the occipital region versus the temporal. One explanation for the higher N1/P1 amplitudes in VR HMD may be related to the relatively more controlled environment in this modality (VR screen display expanded to fit the full field of view with a black void in the periphery) compared to CS (real life laboratory setting with various peripheral objects such as a table and walls). The higher N1/P1 components in VR HMD compared to CS may be related to differences in attention levels between these two screen environments. Higher amplitude anterior N1 and posterior P1 components are associated with elevated early attentional processes (Hillyard and Anllo-Vento 1998; Woodman 2010; Hinojosa et al. 2015). Increases in the focus of attention in the visual field are thought to lead to an increase in the anterior N1 amplitude (Rugg et al. 1987; Luck et al. 2000). It may be the case that in the VR HMD environment there is a restricted field of view compared to CS, and participants thereby have increased focus upon the presented stimuli leading to higher amplitude N1/P1 components.

Another possibility for the higher amplitude N1/P1 values in VR HMD could be related to light intensity (a low level feature of the visual stimuli) which has been shown to affect early N1/P1 ERP components and influence perception of stimuli (Hillyard and Kutas 1983; Johannes et al. 1995; Wijers et al. 1997; Busch et al. 2004; Alorda et al. 2007; Lakens et al. 2013; Schettino et al. 2016; Schindler et al. 2018). Since the intensity of light during the presentation of *n*-back trials for CS and VR HMD was similar (< 0.5 lx difference), a change in the light intensity between the two environments is not likely to explain the higher amplitude N1/P1 components in VR HMD. Our results suggest that researchers investigating N1/P1 components could consider utilising VR HMD as N1/P1 waveforms, based upon our *n*-back task results, are more discernible in amplitude compared to CS.

We cannot exclude the possibility that there could be low level features which could account for the N1/P1 component differences between CS and VR HMD. For example, the size of the *n*-back letter stimuli presented may not have been the same for CS and VR HMD despite our attempts for each participant to have similar screen experiences in both environments (VR HMD lens adjustments, proportional mirroring and adjustments in the parameters for size/depth). Ideally, the size of the letters presented in VR HMD should be measured, and unlike for CS, we cannot identify a technically feasible method to measure the size of the letter stimuli presented in the VR HMD screen. Other low levels features which could account for the differences in N1/P1 components between the two environments may be related to the screen type (LCD versus OLED which could impact upon levels of contrast), screen resolutions and refresh rates.

The anterior N1 and posterior P1 ERP components appear to have a close temporal coincidence in the desktop CS environment (see Fig. 4) which may reflect similar dipole generators (Hillyard and Anllo-Vento 1998; Joyce and Rossion 2005). For the VR HMD environment we also found a close temporal coincidence for the anterior N1 and posterior P1 components. Further investigation using source localisation techniques could reveal the localisation of the neuronal sources for the CS and VR HMD environments.

In our group of participants, physical discomfort was not reported using the HTC Vive system. In contrast, discomfort and pain were reported in the study by Tauscher et al. (2019) which they attributed to the physical pressure placed upon EEG electrodes of the headcap from the overlying holding strap of the HTC Vive VR HMD. One likely explanation for this difference between the studies is that we used an EEG headcap, where the electrodes are encased in a soft sponge material (Sleepcap, Spes Medica), whereas Tauscher et al. (2019) utilised a headcap with electrodes encased in a hard plastic surround. Although Tauscher et al. (2019) was able to overcome participant discomfort by making cut-outs in the HTC Vive strap, we suggest that it may instead be more practical and comfortable when combining EEG headcaps with VR HMD systems to utilise headcaps with electrodes encased in soft materials to absorb pressure from the overlying holding strap of the VR HMD system. In addition, since the EEG headcap used in our investigation has all the wires arising from each electrode located internally within the cap, this would be advantageous in terms of the practicality of donning the VR HMD system, keeping it unmodified and minimising EEG artifacts arising from electrode wire disturbance.

We used an experimental design in our study, where participants donned the same EEG headcap (without removal) for the CS and VR HMD experimental sessions which were completed within ~ 25 min of each other. This design minimises any participant discomfort from wearing the relatively bulky HTC Vive system, and potential differences in electrode location and ERP responses to stimuli as may result if long periods of time have lapsed such as undertaking CS and VR HMD experimental sessions on different days. None of our participants reported tiredness and discomfort when undergoing data acquisition using the EEG/HTC Vive system combination. In contrast, Rupp et al. (2019) mentioned discomfort and neck strain in some participants and attributed this to the VR HMD exerting pressure on the face due to its weight and bulk. The discomfort may have been due to the relatively longer experimental session (~ 50 min) in the investigation by Rupp et al. (2019) versus our shorter session (~ 25 min). Future studies should consider the weight of the VR HMD, and to use lighter weight VR HMDs for longer duration protocols to minimise participant discomfort.

In our current investigation, participants were required to look at each presented stimulus at the centre of the screen and to refrain from making head movements during EEG recordings whilst wearing the HTC Vive VR HMD to minimise eye movement and head movement-induced artifacts. Given the immersive multidimensional nature of a VR HMD system, it would be desirable to acquire EEG data during stimulus presentations, where the participant is able to orientate the head/eyes to any location in the visual field. Tauscher et al. (2019) undertook a comparative investigation of recording ERP visual oddball task responses both without participant head motion (static) and with horizontal and vertical head motions using an EEG system with acquisition electronics mounted at the back of the head. The quality of the ERP waveforms was found to be substantially reduced by horizontal, and especially by vertical, head motions in VR HMD compared to participants looking at a computer screen whilst maintaining a fixed position.

To minimise movement-induced artifacts in the EEG signals, we did not make use of the associated HTC Vive hand controllers as an input method for participants to select target stimuli in our ERP investigation. Hand controllers with motion tracking have been utilised in a visual *n*-back task study conducted by Tremmel et al. (2019) using the HTC Vive system, where participants used their dominant hand to place target/non-target balls upon a receptacle. However, an ERP analysis approach was not employed by these authors, instead they used a power spectral analysis of the various EEG frequency bands. It would be valuable in future studies of VR HMD and ERP if the motion activity and button press responses of hand controllers could be time stamped onto the EEG recording.

In our investigation we mirrored, using Bigscreen Beta software, the desktop CS which was running PEBL presentation software to enable time-locked delivery of each stimulus. A similar setup was used in the study by Rupp et al. (2019) who utilised an HTC Vive VR HMD with an EEG system and demonstrated the successful feasibility of acquiring ERPs in response to visual attention and memory tasks using Unreal Engine 4 software for time-locked stimulus delivery. In our study, we duplicated the contents of the desktop CS screen on the HTC Vive, whereas Rupp et al. (2019) generated a virtual computer screen placed upon a virtual table. Notwithstanding this difference, our ERP waveforms with the HTC Vive were similar in profile to those found by Rupp et al. (2019); the peak amplitude of the P3 component in our study and that reported by Rupp et al. (2019) were both in the range 5–10 μV with ~ 400 ms for the peak latency.

Adverse effects termed cybersickness, simulator sickness, motion sickness, nausea, and disorientation have been reported in participants using VR HMD systems (Moss and Muth 2011; Arafat et al. 2018; Saredakis et al. 2020; Heo and Yoon 2020). Such adverse symptoms may arise due to various factors including those related to the content presented in VR, the amount of visual movements, participant locomotion, amount of time spent in the VR environment, the demographic user profiles and the technical specifications of the hardware used (see Kourtesis et al. 2019; Saredakis et al. 2020). Only one participant (from a total of 21) in our visual ERP investigation reported feeling the sensation of motion sickness when wearing the HTC Vive system. In their ERP study, Harjunen et al. (2017) reported a single participant (from a total of 12) who experienced nausea using the Oculus Rift DK2. Other recent visual ERP investigations have not reported motion sickness/nausea in participants when using VR HMD sets (Tromp et al. 2018; Rupp et al. 2019; Tauscher et al. 2019; Stolz et al. 2019; Du et al. 2019; Spapé et al. 2019; Sun et al. 2019). We recommend that all EEG studies which integrate with VR HMD systems explicitly report the number of participants who experience motion sickness/nausea including if none was experienced. Given the rapid pace of improvements in system technical specifications including higher display resolutions and faster image refresh rates (see Kourtesis et al. 2019) the incidence of reported motion sickness/nausea could be expected to be reduced in future studies using VR HMD systems.

## Conclusion

We have demonstrated the experimental feasibility and successful acquisition of visual electrophysiological ERPs in response to a cognitive working memory task whilst donning a VR HMD over an EEG headcap without the need to make any modifications or customisations. The ERP waveforms obtained using the VR HMD followed a similar time course to those acquired in the CS environment. The P3 mean and peak amplitude components obtained in the VR environment were not significantly different from those acquired in the CS environment. However, we did find significant differences between the responses seen with VR HMD compared to those for CS for the early ERP components. The N1 component was significantly higher in mean amplitude and peak amplitude for the VR HMD environment compared to CS for electrodes at the frontal region. For VR HMD, there were significantly higher P1 mean and peak amplitudes at the occipital region compared to the temporal region but not for CS. Based upon the *n*-back task, our results indicate that researchers can take advantage of VR HMD systems to acquire ERP waveforms from participants undertaking cognitive tasks in experimental brain research investigations. Although requiring further study, the higher amplitude N1/P1 components found in our results using VR HMD indicates the potential usefulness of this modality in the investigation of early ERP components.

## Data Availability

Data will be made available on reasonable request.
